# A study on risk factors of acute ischemic stroke after radiofrequency ablation in patients with atrial fibrillation

**DOI:** 10.3389/fcvm.2026.1791857

**Published:** 2026-04-29

**Authors:** Chunjie Wen, Ying Zhong, Meng Wang, Shengbo Jiang, Xiaoxiao Yin, Rui Liu, Bin Yu, Jifang Cheng

**Affiliations:** 1 Cardiovascular Intervention Center, The Second Affiliated Hospital, Zhejiang University School of Medicine, Hangzhou, Zhejiang, China; 2 Endoscopy Center, The Second Affiliated Hospital, Zhejiang University School of Medicine, Hangzhou, Zhejiang, China

**Keywords:** acute ischemic stroke, atrial fibrillation, prognosis, radiofrequency catheter ablation, risk factors

## Abstract

**Objective:**

To explore the risk factors for acute ischemic stroke (AIS) in patients with atrial fibrillation (AF) after radiofrequency catheter ablation (RFCA).

**Methods:**

A total of 498 patients with AF who underwent RFCA treatment at the Second Affiliated Hospital of Zhejiang University School of Medicine from June 2022 to June 2024 and had 1-year follow-up data were retrospectively selected as the research subjects. The patients were divided into the Non-AIS group (*n* = 450) and the AIS group (*n* = 48) based on whether AIS occurred during the follow-up period. General and clinical data of the patients were collected. Logistic regression was used to analyze the risk factors for AIS following RFCA in AF patients, and the regression model was validated.

**Results:**

There were no statistically significant differences in general information between the Non-AIS group and the AIS group (*P* > 0.05); there were statistically significant differences in the incidence of history of cardiac insufficiency (CI), types of AF, and platelet/lymphocyte ratio (PLR) between the Non-AIS group and the AIS group (*P* < 0.05). The forest plot of the multivariate Logistic regression model showed that history of CI, persistent AF, and PLR were independent risk factors for AIS after RFCA within 1 year (*P* < 0.05). The individual and combined areas under the curve were 0.817 (0.756–0.879), 0.579 (0.486–0.673), 0.644 (0.565–0.724), and 0.852 (0.798–0.905), respectively. The cut-off value, sensitivity, and specificity of the combined receiver operating characteristic (ROC) curve were 0.287, 0.813, and 0.758, respectively. The calibration curve and clinical decision curve showed that the Logistic regression model had good accuracy.

**Conclusion:**

The overall incidence of AIS within 1 year after RFCA in patients with AF is relatively low (9.64%). History of CI, persistent AF, and PLR are independent risk factors for AIS after RFCA within 1 year. Preoperative screening and strict control of indications can help reduce the occurrence of AIS after RFCA.

## Introduction

Atrial fibrillation (AF) is a common and complex arrhythmia that holds a significant position in the field of cardiovascular diseases (1). In recent years, with the rapid acceleration of the global aging process, the incidence of AF has shown a worrying annual increase trend. Globally, the number of AF/Atrial flutter (AFL) cases in 2021 was approximately 4.5 million, and according to the data from China in 2021, the number of people suffering from AF was 10.78 million, with 920,000 new cases and 60,000 deaths (2, 3). According to statistical data, the prevalence of AF in the elderly population is significantly higher than that in other age groups, and it continues to rise with the increase in age (4). This disease not only imposes a heavy burden on the medical system, but also poses a serious threat to the quality of life and health of a large number of patients. The main treatment methods for AF include drug therapy, device therapy, radiofrequency catheter ablation (RFCA), and surgical treatment (5, 6). Among them, RFCA has become an extremely effective method for controlling heart rhythm (7, 8). Restoring sinus rhythm through RFCA significantly alleviates AF-related symptoms and improves patients’ physical capacity and overall quality of life, facilitating a return to normal activities (9).

However, although the RFCA technology has made significant progress in recent years, the occurrence of postoperative complications remains an important issue that cannot be ignored in clinical practice. Among the various possible complications, acute ischemic stroke (AIS) is one of the most serious neurological complications (10). In patients who experience AIS after RFCA, not only does it lead to further deterioration of their already fragile physical condition, but it also significantly prolongs the hospital stay (11). Additionally, AIS may cause permanent neurological deficits in patients, such as hemiplegia, aphasia, and cognitive impairment, which will seriously affect the patients’ ability to take care of themselves and greatly reduce their quality of life (12, 13). Studies have consistently shown that patients with AF face a significantly elevated risk of AIS, with a risk four to five times higher than the general population, accounting for an estimated 15%–20% of all ischemic strokes (14, 15). This risk varies depending on known factors, including advanced age, hypertension, diabetes, prior stroke or transient ischemic attack, and heart failure (16).

However, there are few reports on the risk factors for AIS after RFCA. Based on this, this article conducts a retrospective analysis of the clinical data of AF patients who received RFCA to explore the risk factors for postoperative AIS, aiming to provide scientific evidence for accurately identifying high-risk patients in clinical practice, formulating individualized prevention strategies, and optimizing postoperative management, thereby effectively reducing the incidence of AIS after RFCA and improving the prognosis and quality of life of patients.

## 1 Research subjects and methods

### 1.1 Research subjects

A retrospective consecutive selection was made of 498 AF patients who received RFCA treatment at the Second Affiliated Hospital of Zhejiang University School of Medicine from June 2022 to June 2024 and had complete 1-year follow-up data. The patients were divided into the Non-AIS group (*n* = 450) and the AIS group (*n* = 48) based on whether AIS occurred during the follow-up period.

Inclusion criteria: ① age≥18 years; ② diagnosis of AF, including paroxysmal and persistent AF (17); ③ undergoing their first RFCA for AF; ④ having complete clinical and follow-up data; Exclusion criteria: ① undergoing RFCA for arrhythmias other than AF; ② having advanced tumors, hematological diseases, respiratory failure, or other severe end-stage diseases; ③ presenting with critical or urgent conditions, unstable mental state, or confusion; ④ having comprehension, hearing, or reading impairments; ⑤ having missing clinical data or follow-up data; ⑥ being unable to discontinue anticoagulant therapy due to complications; This study has been reviewed and approved by the Medical Ethics Committee of the Second Affiliated Hospital of Zhejiang University School of Medicine [Ethical Number: 2025-51-02].

### 1.2 Diagnostic criteria

The AF diagnostic criteria are in accordance with the latest guidelines for the diagnosis and treatment of AF issued by the American Heart Association, the American College of Cardiology, and the American Heart Rhythm Society in 2023. Paroxysmal AF is defined as AF lasting less than 7 days, while persistent AF is defined as AF lasting 7 days or more (17).

The diagnosis of AIS conforms to the diagnostic criteria of the 2023 American Heart Association/American Stroke Association secondary prevention guidelines for AIS onset. Focal neurological deficits lasting more than 24 h or having imaging evidence of cerebral infarction, and focal neurological symptoms or signs that persist for no more than 24 h are diagnosed as AIS (18).

The history of cardiac insufficiency (CI) was defined as a prior diagnosis of heart failure, a left ventricular ejection fraction ≤50% on admission echocardiography, or a New York Heart Association (NYHA) functional class of II or higher (19).

### 1.3 Data collection

All baseline clinical data of the enrolled patients were collected through the hospital's electronic medical record system. The data were independently extracted by two researchers, and a cross-check was conducted after extraction. In case of any disagreement, a third researcher would make the final decision.

Collect the gender, age, smoking history, drinking history, body mass index (BMI), diabetes history, hypertension history, history of CI, hyperlipidemia history, type of AF (Paroxysmal AF: Terminates spontaneously or is terminated through intervention within 7 days; Persistent AF: Duration of attack >7 days), number of surgeries, surgical approach, CHA_2_DS_2_-VASc, rivaroxaban usage, platelet/lymphocyte ratio (PLR), creatinine (CREA), epidermal growth factor receptor (EGFR), high-density lipoprotein cholesterol (HDL-C), and total cholesterol (TC) value of all patients. All the data were logically verified before being entered to ensure the accuracy and completeness of the information.

### 1.4 Collection of follow-up data

All patients have complete follow-up records. The follow-up period is 12 months after the surgery. The follow-up methods include outpatient re-examination, telephone interviews, and review of hospital medical records. The regular follow-up points are set at 1 month, 3 months, 6 months, and 12 months after the surgery; for patients who report neurological deficits during this period, urgent follow-up evaluations will be arranged at any time.

The data collected during the follow-up period include: patient symptoms, electrocardiogram results, and 24-h ambulatory electrocardiogram results to assess the control of heart rhythm, as well as the use and compliance of anticoagulant drugs. The primary endpoint of observation is the occurrence of AIS within 1 year after the surgery. The diagnosis of AIS conforms to the diagnostic criteria of the 2023 American Heart Association/American Stroke Association “Secondary Prevention Guidelines for AIS” (18). All suspected stroke cases are confirmed by neurologists based on the imaging results of head CT or MRI. For patients who are lost to follow-up, the reasons for loss and the last contact time are recorded.

### 1.5 RFCA treatment method

All patients underwent the surgery in a fasting and sedated state. A coronary venous sinus electrode was inserted through the left subclavian vein, and a transseptal puncture was performed through the right femoral vein. During the operation, heparin was used to maintain the activated clotting time within 250–300 s. Left atrial reconstruction was performed under the guidance of a three-dimensional electroanatomical mapping system, and radiofrequency ablation was carried out using 30–35 W, 45 °C, and 17 mL/min saline infusion. Each ablation point was considered successful when the local potential decreased by more than 70% or when the discharge lasted for 30 s. All patients underwent ablation based on the bilateral pulmonary vein electrical isolation strategy. The end point of paroxysmal AF ablation was the verification by the pulmonary vein ring mapping electrode that all pulmonary veins had achieved electrical isolation. For patients with non-paroxysmal AF, after completing pulmonary vein isolation, if voltage mapping during the operation reveals the presence of a low-voltage area in the left atrium (bipolar voltage<0.5 mV) or spontaneous/induced atrial tachycardia, additional substrate modification will be performed based on the surgeon's judgment, including complex fragmented potential ablation, linear ablation of the left atrial roof line or the isthmus of the mitral valve line. All patients were treated by the same surgical team.

### 1.6 Postoperative anticoagulation therapy

All patients were given rivaroxaban at a dose of 15–20 mg for anticoagulation until 3 months after the surgery. Three months after the surgery, two senior cardiologists with over ten years of experience jointly reassessed the anticoagulation regimens for all patients. The assessment was based on the CHA_2_DS_2_-VASc scoring system (20), and if a patient's CHA_2_DS_2_-VASc score was ≥2, anticoagulation treatment was required.

### 1.7 Statistical analysis

Data analysis was conducted using SPSS 27.0 software (IBM Corporation, Armonk, NY, USA). All variables were subjected to normality and homogeneity of variance tests. For numerical variables that followed a normal distribution and had homogeneous variances, the mean ± standard deviation $\left(\bar{x}\pm s\right)$ was used for description, and *t*-tests were employed for comparisons between groups; for non-normal distribution of measurement data, the median and interquartile range were used for representation, and the Wilcoxon test was applied for comparisons between groups. Count data were expressed as frequencies and percentages, and *χ*^2^ tests were used for comparisons; significant factors from the univariate analysis were included in the multivariate Logistic regression model, and the stepwise regression method was used to determine the independent risk factors; Logistic regression model forest plots, receiver operating characteristic (ROC) curves, calibration curves, and clinical decision curves were drawn using R 4.4.x. *P* < 0.05 was considered statistically significant.

## 2 Results

### 2.1 Comparison of general information

A comparison of the general data of the two groups of patients showed that there were no differences in all basic data between the Non-AIS group and the AIS group (*P* > 0.05), indicating that the two groups were comparable. See Table 1.

**Table 1:** Comparison of general information.

Index	Non-AIS (*n* = 450)	AIS (*n* = 48)	*t/χ* ^2^	*P*
Gender/*n* (%)			0.478	0.489
Male	316 (70.22)	36 (75.00)		
Female	134 (29.78)	12 (25.00)		
Age (year, $\bar{x}\pm s$)	59.09 ± 9.90	60.94 ± 8.28	−1.237	0.217
BMI (kg/m², $\bar{x}\pm s$)	21.00 ± 4.07	21.19 ± 4.65	−0.306	0.759
Smoking history/*n* (%)			1.126	0.289
Yes	270 (60.00)	25 (52.08)		
No	180 (40.00)	23 (47.92)		
Allergy history/*n* (%)			0.045	0.832
Yes	362 (80.44)	38 (79.17)		
No	88 (19.56)	10 (20.83)		
Hypertension/*n* (%)			0.177	0.674
Yes	220 (48.89)	25 (52.08)		
No	230 (51.11)	23 (47.92)		
Diabetes/*n* (%)			2.116	0.146
Yes	67 (14.89)	11 (22.92)		
No	383 (85.11)	37 (70.08)		
Hyperlipidemia			1.131	0.717
Yes	222 (49.33)	25 (52.08)		
No	228 (50.67)	23 (47.92)		

### 2.2 Comparison of clinical data

There were no significant differences in history of coronary heart disease (CHD), number of surgeries, operative route, CHA_2_DS_2_-VASc, rivaroxaban usage, EGFR, HDL-C, TC and CREA between Non-AIS and AIS (*P* > 0.05); There were significant differences in the type of AF, PLR, and history of CI indicators. Compared with Non-AIS, had a higher proportion of persistent AF, significantly higher PLR, and more CI history (*P* < 0.05). See Tables 2, 3.

**Table 2:** Comparison of clinical data between the two groups of patients

Index	Non-AIS (*n* = 450)	AIS (*n* = 48)	*t/χ* ^2^	*P*
Type of AF/*n* (%)			14.583	<0.001
Persistence	189 (42.00)	34 (70.83)		
Paroxysmal	261 (58.00)	14 (29.17)		
History of CHD/*n* (%)	40 (8.89)	8 (16.67)	3.013	0.083
History of CI/*n* (%)	13 (2.89)	9 (18.75)	25.842	<0.001
Number of surgeries/*n* (%)			2.383	0.123
1	414 (48.89)	41 (52.08)		
≥2	36 (51.11)	7 (47.92)		
Operative route/*n* (%)			2.484	0.115
Femoral vein	317 (70.44)	39 (81.25)		
Other	133 (29.56)	9 (18.25)		
CHA_2_DS_2_-VASc (score)	1.05 ± 0.23	1.13 ± 0.61	−1.827	0.068
Rivaroxaban (mg)	30.09 ± 8.02	30.91 ± 10.89	−0.648	0.517

**Table 3:** Comparison of serum indicators between the two groups of patients

Index	Non-AIS (*n* = 450)	AIS (*n* = 48)	*t/χ* ^2^	*P*
PLR (year, $\bar{x}\pm s$)	166.21 ± 39.35	217.78 ± 40.28	−8.594	<0.001
CREA (kg/m², $\bar{x}\pm s$)	69.89 ± 8.18	67.84 ± 8.30	1.644	0.101
EGFR (mL·min^−1^/1.73m^−2^, $\bar{x}\pm s$)	119.56 ± 10.31	120.24 ± 10.39	−0.431	0.666
HDL-C (mmol/L, $\bar{x}\pm s$)	0.80 ± 0.05	0.79 ± 0.06	0.833	0.408
TC (mmol/L, $\bar{x}\pm s$)	4.81 ± 0.51	4.68 ± 0.53	1.722	0.086

### 2.3 Logistic regression analysis

The analysis of risk factors for AIS revealed that Type of AF (95% CI: 1.335–5.919), PLR (95% CI: 1.022–2.041), and history of CI (95% CI: 1.225–11.313) were independent risk factors for AIS in AF patients after RFCA (*P* < 0.05). The forest plot of the Logistic regression model showed that type of AF, PLR, and history of CI were all independent risk factors for AIS in AF patients after RFCA, and had the value for predicting AIS. See Table 4.

**Table 4:** Logistic regression analysis of the occurrence of AIS

Index	*β*	S.E.	Wald *χ*^2^	*P*	OR		95% CI
Type of AF	1.034	0.380	7.407	0.006	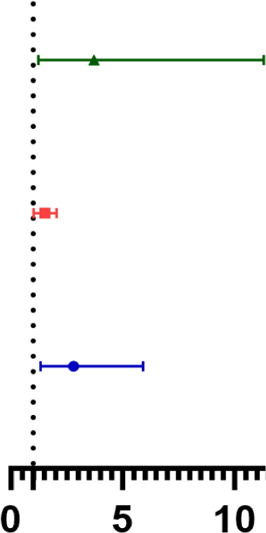	2.812	1.335–5.919
PLR	0.031	0.005	45.659	<0.001		1.532	1.022–2.041
CI history	1.314	0.567	5.372	0.020		3.723	1.225–11.313

### 2.4 ROC analysis

**Figure 1:**
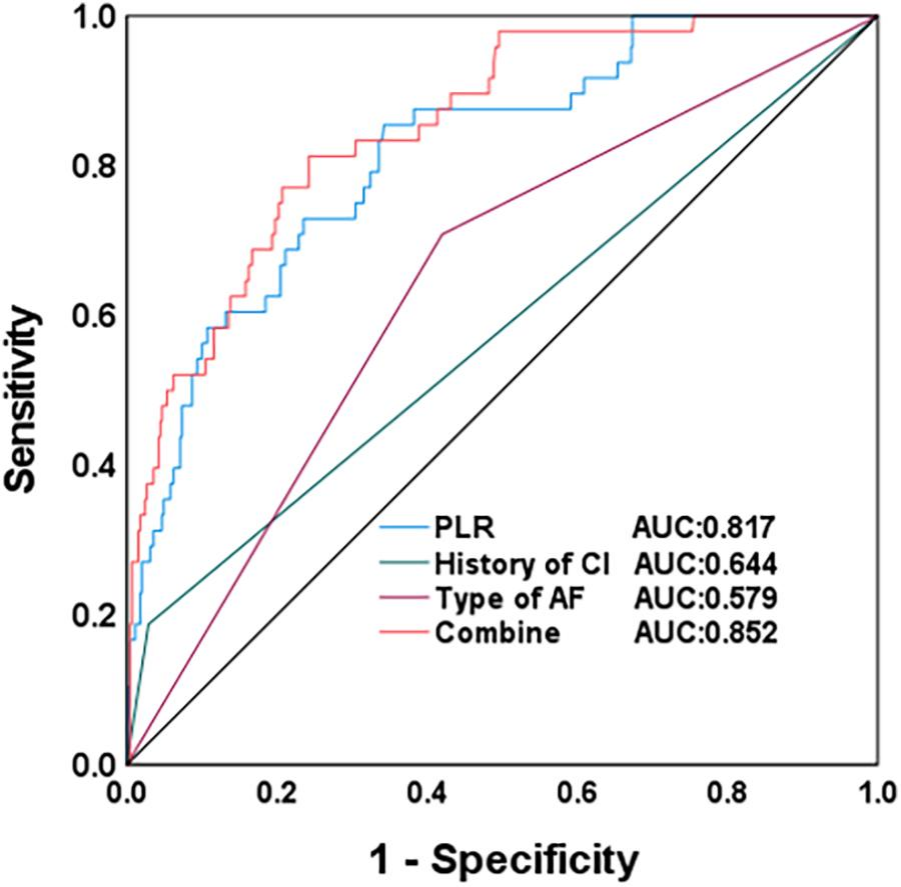
ROC curve of AIS risk factors.

The ROC curve analysis indicated that the PLR value, the history of CI, and the type of AF have extremely high predictive efficacy for the risk of AIS in patients with AF after RFCA. The individual and combined areas under the curve were 0.817 (0.756–0.879), 0.579 (0.486–0.673), 0.644 (0.565–0.724), and 0.852 (0.798–0.905), respectively. The ROC curve analysis revealed that the three risk factors all had certain predictive value for the occurrence risk of AIS after radiofrequency ablation, but the predictive efficacy varied. The area under the curve (AUC) of the combined prediction reached 0.852, which was significantly higher than the predictive efficacy of any single factor, indicating that the combined model can more comprehensively reflect the overall risk status of patients and has higher predictive accuracy. The cut-off value, sensitivity, and specificity of the combined ROC curve were 0.287, 0.813, and 0.758, respectively. This result indicates that when the combined prediction probability is greater than 0.287, the risk of AIS occurring after the surgery for the patient significantly increases. See Table 5 and Figure 1.

**Table 5:** ROC analysis of the occurrence of AIS

Index	AUC	Cutoff value	Sensitivity	Specificity	95% CI
PLR	0.817	180.825	0.854	0.658	0.756–0.879
History of CI	0.579	0.500	0.188	0.971	0.486–0.673
Type of AF	0.644	0.500	0.708	0.580	0.565–0.724
Combine	0.852	0.287	0.813	0.758	0.798–0.905

### 2.5 Calibration curve and clinical decision curve analysis

**Figure 2:**
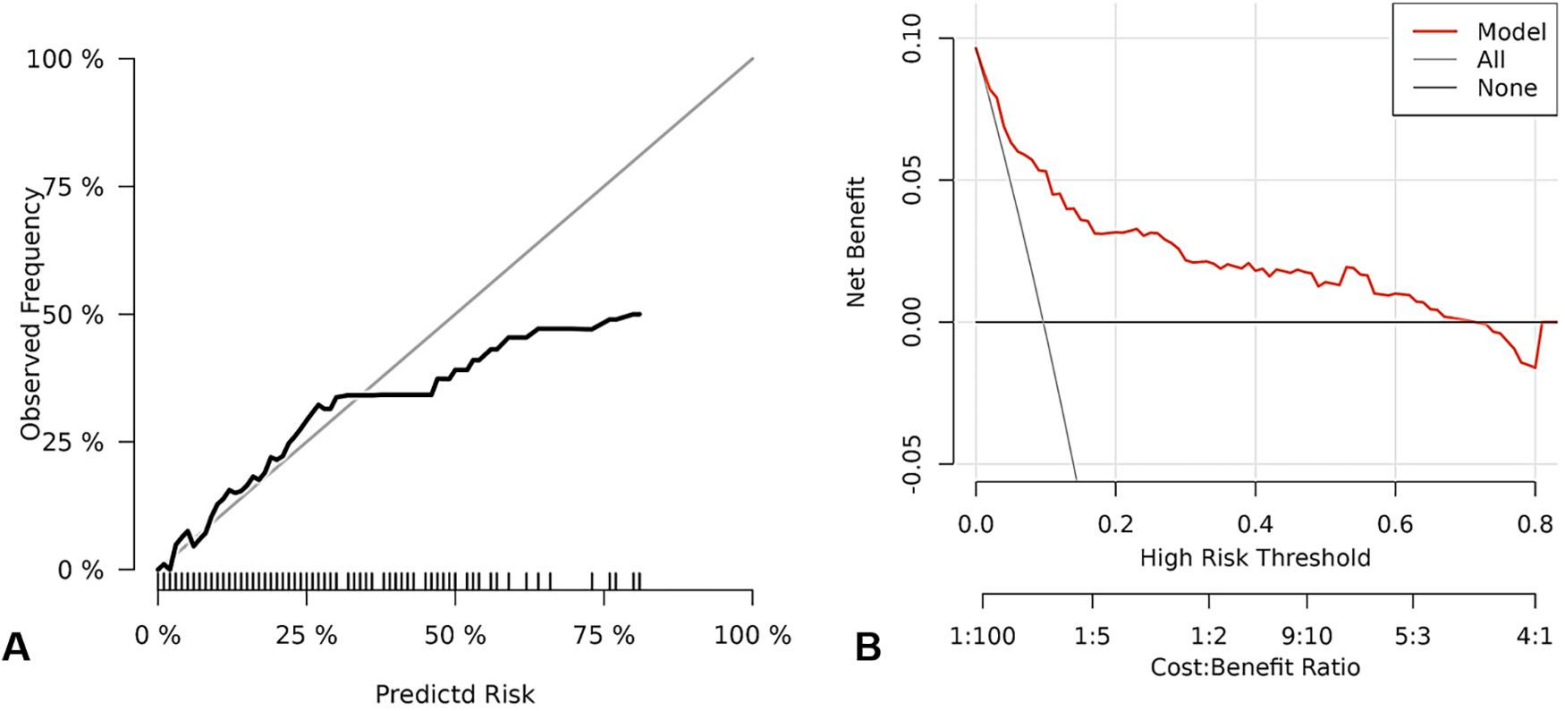
The calibration curve of AIS risk factors and the clinical decision curve. Note: **(A)** represents the calibration curve; **(B)** represents the clinical decision curve.

The calibration curve analysis revealed that this predictive model exhibited excellent accuracy and consistency in predicting the probability of AIS occurrence. As shown in Figure 2A, the model constructed based on three risk factors: type of AF, PLR, and history of cardiac dysfunction, demonstrated a high degree of concordance between the predicted probability of AIS occurrence and the actual observed incidence of AIS. The calibration curve of the model was close to the ideal diagonal reference line, indicating that there was no significant systematic deviation of the model within the entire predicted probability range. After calculation, the average absolute error between the model's predicted values and the actual observed values was 0.066, further confirming that the model has good calibration and can accurately reflect the actual risk of AIS in AF patients after radiofrequency ablation.

Clinical decision curve analysis was used to evaluate the clinical practical value of this predictive model. As shown in Figure 2B, when the risk threshold probability was set within a wide range of 1%–71%, the predictive model constructed based on type of AF, PLR, and history of cardiac dysfunction demonstrated a positive net benefit in guiding clinical decisions. This means that within this threshold range, using this model to identify high-risk patients and taking corresponding intervention measures brought clinical benefits that outweighed the harm caused by unnecessary interventions due to misjudgment. This result indicates that this predictive model not only has good discrimination and calibration, but also has potential clinical application value, which can provide a reference basis for clinicians to identify high-risk patients after surgery and formulate individualized monitoring and prevention strategies.

## Discussion

Currently, RFCA therapy is one of the most commonly used treatments for patients with AF, and its core objective is to restore and maintain sinus rhythm for a long term (21). RFCA treatment can improve the clinical symptoms of AF patients, enhance their quality of life, and potentially reduce the risk of long-term thromboembolic events (22). However, during the process of achieving rhythm control with RFCA, the prevention and treatment of perioperative and postoperative complications remain important challenges in clinical practice (23). Among these complications, AIS is one of the most serious neurological adverse events (24). The functional impairments caused by AIS complications will reduce patients’ ability to perform daily self-care tasks and have a significant negative impact on their quality of life (10). This study found through retrospective analysis that the overall incidence of AIS within one year after RFCA in AF patients was 9.64%. Furthermore, it was further confirmed that persistent AF, a higher PLR, and previous cardiac dysfunction were independent risk factors for AIS within one year after surgery. This indicates that for patients with AF who are scheduled for RFCA, preoperative assessment should not only focus on the success rate of the surgery and the type of arrhythmia, but also screen their cardiac function status, the form of persistent AF, and PLR indicators. This finding is of great significance for optimizing the stroke prevention strategy during the perioperative period of RFCA for AF patients. It proposes early warning indicators for the risk of AIS after ablation, and under its guidance, it is expected to maximize the benefits of RFCA while minimizing the associated risk of AIS.

In previous studies, it was found that patients with AF who had a history of cardiac dysfunction often had more cardiac-related and thromboembolic complications after surgery (25). Studies have shown that persistent AF has a negative impact on both the prognosis and quality of life of patients (26). Furthermore, PLR can reflect the “high inflammation-high coagulation” state of the body. An elevated PLR indicates an imbalance between inflammatory activation and immune response in the body, and is an important indicator for assessing the thrombotic state (27). In the case of RFCA surgery, a high PLR level can provide a reference basis for perioperative risk stratification and intervention strategies (28). The results of this study have shown that the overall incidence of AIS within one year after RFCA was 9.64%, and it was found that there were statistically significant differences between the AIS group and the Non-AIS group in terms of history of heart failure, type of AF, and PLR (*P* < 0.05). The overall incidence of AIS within one year after RFCA was relatively low, which was similar to the incidence of 10.10% in previous literature (29). The above statement indicates that the possible risk factors for AIS occurrence within one year after RFCA surgery are a history of heart dysfunction, persistent AF, and high levels of PLR.

Previous studies have confirmed that CI is an important risk factor for thromboembolic events in patients with AF (25, 30). The pathogenesis mechanism mainly involves the following aspects. Firstly, CI leads to a decline in left ventricular systolic function, causing a reduction in blood flow velocity within the heart chambers and blood stasis, creating hemodynamic conditions for thrombosis (31); Secondly, CI is often accompanied by excessive activation of the sympathetic nervous system and the renin-angiotensin-aldosterone system, which can further aggravate endothelial dysfunction and platelet activation (30); Moreover, CI patients often have a higher inflammatory load. There is a complex interaction between inflammation and the coagulation system, jointly promoting the formation of a hypercoagulable state (25). The results of this study are consistent with the above mechanisms, further confirming that even in patients with heart dysfunction who receive RFCA treatment, their inherent tendency to form thrombosis has not been completely eliminated. Postoperatively, there is still a need to be highly vigilant against the occurrence of AIS.

The type of AF is another important independent predictor. In this study, patients with persistent AF had a significantly higher risk of AIS after surgery than those with paroxysmal AF, which is consistent with the conclusions of previous studies (26). From a pathological perspective, persistent AF has a multi-faceted impact on thrombosis (32). Patients with persistent AF often have more significant atrial enlargement and myocardial fibrosis, which are structural changes that not only serve as the substrate for maintaining AF but also provide an attachment basis for the formation of AIS (33). Additionally, compared to paroxysmal AF, the effective atrial contraction function of patients with persistent AF is almost completely lost, and the blood flow velocity in the left atrial appendage significantly slows down, resulting in more severe local blood stasis (34). Furthermore, the surgical strategies for patients with persistent AF undergoing RFCA are often more complex. In addition to pulmonary vein isolation, additional matrix modification is often required, prolonging the operation time and increasing the number of atrial catheter operations, which may increase the risk of microthrombus detachment (32). Therefore, for patients with persistent AF undergoing RFCA, even if the surgery is successful immediately, their inherent thrombo-preventive state still exists, and postoperative measures for AIS prevention still need to be strengthened.

PLR, as a comprehensive biomarker reflecting the relationship between systemic inflammation levels and coagulation status, has received extensive attention in the prediction of cardiovascular disease risks in recent years (28). This study found that an elevated preoperative PLR is an independent risk factor for postoperative AIS, and its sole prediction has an AUC of 0.817, demonstrating good discriminatory ability. The pathophysiological basis of PLR lies in its integration of dual information of inflammatory activation and immune regulation (27). On one hand, platelets, as the core participants in inflammatory cells and thrombosis, their increased quantity and enhanced activation can directly promote thrombosis; on the other hand, the reduction of lymphocytes reflects the immune stress state and imbalance of inflammation regulation in the body, and this “high inflammation-low immunity” state is closely related to adverse cardiovascular events (35). During the perioperative period of RFCA, surgery itself can induce local and systemic inflammatory responses, and for patients with already high inflammatory levels before surgery, this cumulative effect may further exacerbate the hypercoagulable state and increase the risk of thrombotic events (36). Therefore, as a simple, inexpensive, and repeatable laboratory indicator, PLR is expected to become a powerful tool for preoperative risk stratification in RFCA.

Furthermore, the study found that although the AUC of history of CI alone was only 0.579, and the AUC of AF type alone was 0.644, the discrimination ability was relatively limited. However, when the three factors were jointly predicted, the AUC increased to 0.852 (95% CI: 0.798–0.905), with a sensitivity of 81.3% and a specificity of 75.8%, which was significantly better than any single indicator. This result suggests that the occurrence of AIS is the result of the combined action of multiple factors, and a single factor is difficult to fully reflect the overall risk status of the patient (37). The advantage of the combined prediction model lies in integrating multi-dimensional information reflecting cardiac structure and function, atrial electrical mechanical characteristics, and systemic inflammatory and coagulation status, which more comprehensively captures the pathophysiological basis of postoperative AIS risk. The calibration curve analysis showed that the average absolute error between the predicted probability and the actual incidence rate was only 0.066, indicating that the model has good calibration. The clinical decision curve analysis further confirmed that when the risk threshold probability was within a wide range of 1%–71%, clinical decision-making based on these three factors could achieve positive net benefit. This means that using this model to identify high-risk individuals and taking targeted intervention measures has clinical benefits exceeding the harm caused by unnecessary intervention due to misjudgment.

The results of this study are highly consistent with previous literature (36). This consistency may stem from several reasons. Firstly, CI, and persistent AF reflect the core pathophysiological mechanisms of thrombosis, including blood stasis and abnormal vascular walls (32). Secondly, PLR, as a stable biomarker reflecting systemic inflammation and hypercoagulable state, has a relatively standardized detection method and good comparability among different studies (38). Building upon previous research, this study further integrates these three indicators reflecting different dimensions into a combined predictive model, providing a more comprehensive assessment tool for the risk of stroke after RFCA.

In terms of clinical significance, the findings of this study provide an important theoretical basis for the perioperative management of RFCA. For patients with CI, persistent AF, and elevated preoperative PLR, they should be regarded as a high-risk group for postoperative AIS. For this group, clinicians can consider adopting more proactive individualized prevention strategies, including optimizing the perioperative anticoagulation regimen, strengthening brain protection measures during surgery, enhancing neurological monitoring after surgery, and exploring the value of new anti-inflammatory adjuvant treatments. Additionally, these patients should also receive more detailed stroke warning education upon discharge from the hospital to improve their ability to recognize related symptoms and their awareness of seeking medical attention.

However, this study still has certain limitations. First, the research design was a single-center retrospective analysis, with a relatively small sample size, which may lead to selection bias and information bias. The extrapolation of the conclusion is limited to a certain extent. Although we controlled some confounding factors through multivariate regression analysis, there may still be unmeasured confounding variables that affect the results. Second, the detection of PLR was based solely on a single blood sample taken before the operation, lacking dynamic monitoring data. As an inflammatory marker, PLR may be affected by acute infections, stress states, or other comorbidities. A single measurement fails to fully reflect the dynamic changes in the body. Third, the follow-up time of this study was only 1 year after the operation. The long-term risk of AIS is still unclear, especially as time progresses, the recurrence of AF may bring new thrombosis risks. Fourth, due to the sample size limitation, a more detailed stratified analysis of different AF subtypes was not conducted. Fifth, this study did not include imaging indicators, which may provide additional predictive value. Future research directions: Conduct large-scale, multi-center, prospective cohort studies, including populations from different regions and races, to improve the generalizability of the research conclusion; extend the follow-up time to assess the long-term risk of AIS and the impact of AF recurrence on stroke risk; establish a multi-dimensional predictive model including clinical characteristics, biomarkers, and imaging indicators to further improve the prediction accuracy; explore individualized intervention strategies based on risk stratification to improve the prognosis of patients; dynamically monitor the changes in inflammatory markers such as PLR during the perioperative period, and clarify the optimal detection timing and intervention threshold.

In conclusion, this study found that the history of CI, the type of persistent AF, and the elevated preoperative PLR were independent risk factors for AIS occurring within one year after radiofrequency ablation in AF patients. The combined prediction model constructed based on these three factors has good discrimination, calibration, and clinical net benefit, and can effectively identify patients at high risk of postoperative AIS. This finding suggests that even if RFCA successfully restores sinus rhythm, the patient's inherent thrombotic state has not been completely eliminated. For patients with CI, persistent AF, and high PLR, more aggressive individualized stroke prevention strategies should be adopted during the perioperative period and long-term postoperative management. Future prospective multicenter studies are needed to further verify the application value of this model and explore the significance of risk stratification-based intervention strategies in improving patient prognosis.

## Data Availability

The raw data supporting the conclusions of this article will be made available by the authors, without undue reservation.

## References

[1] Saleh K Haldar S. Atrial fibrillation: a contemporary update. Clin Med (Lond). (2023) 23(5):437–41. 10.7861/clinmed.2023-23.5.Cardio2 37775166 PMC10541273

[2] Karatela MF Calkins H. The global impact of atrial fibrillation. Arrhythm Electrophysiol Rev. (2025) 14:e28. 10.15420/aer.2025.33 41346409 PMC12673497

[3] Wang Z Shi Z Shen J Zhang S Fang Y Hu W Analysis and comparison of the trends in burden of atrial fibrillation/atrial flutter in China and worldwide from 1990 to 2021 and predictions to 2036 of China. Front Cardiovasc Med. (2025) 12:1540750. 10.3389/fcvm.2025.1540750 40520932 PMC12162966

[4] SM A-K. Atrial fibrillation. Ann Intern Med. (2023) 176(7):Itc97–112. 10.7326/aitc202307180 37429028

[5] Turagam MK Musikantow D Whang W Koruth JS Miller MA Langan MN Assessment of catheter ablation or antiarrhythmic drugs for first-line therapy of atrial fibrillation: a meta-analysis of randomized clinical trials. JAMA Cardiol. (2021) 6(6):697–705. 10.1001/jamacardio.2021.0852 33909022 PMC8082432

[6] O'Keefe EL Sturgess JE O'Keefe JH Gupta S Lavie CJ. Prevention and treatment of atrial fibrillation via risk factor modification. Am J Cardiol. (2021) 160:46–52. 10.1016/j.amjcard.2021.08.042 34583808

[7] Waranugraha Y Tsai CT Lin LY. Index-Guided high-power radiofrequency catheter ablation for atrial fibrillation: a systematic review and meta-analysis study. Curr Cardiol Rep. (2023) 25(11):1397–414. 10.1007/s11886-023-01968-6 37874469

[8] Reddy VY Peichl P Anter E Rackauskas G Petru J Funasako M A focal ablation catheter toggling between radiofrequency and pulsed field energy to treat atrial fibrillation. JACC Clin Electrophysiol. (2023) 9(8 Pt 3):1786–801. 10.1016/j.jacep.2023.04.002 37227340

[9] Mililis P Kariki O Saplaouras A Bazoukis G Dragasis S Patsiotis IG Radiofrequency versus cryoballoon catheter ablation in patients with persistent atrial fibrillation: a randomized trial. J Cardiovasc Electrophysiol. (2023) 34(7):1523–8. 10.1111/jce.15965 37293822

[10] Benali K Khairy P Hammache N Petzl A Da Costa A Verma A Procedure-Related complications of catheter ablation for atrial fibrillation. J Am Coll Cardiol. (2023) 81(21):2089–99. 10.1016/j.jacc.2023.03.418 37225362

[11] Cho MS Lee SR Black-Maier E Jackson KP Friedman DJ Pokorney SD Complications associated with pulsed field ablation vs radiofrequency catheter ablation of atrial fibrillation. Heart Rhythm. (2025) 22(9):2194–200. 10.1016/j.hrthm.2024.10.063 39515491

[12] Migdady I Russman A Buletko AB. Atrial fibrillation and ischemic stroke: a clinical review. Semin Neurol. (2021) 41(4):348–64. 10.1055/s-0041-1726332 33851396

[13] Alhatemi RAJ Sava S. A weighted ensemble approach with multiple Pre-trained deep learning models for classification of stroke. Medinformatics. (2023) 1(1):10–9. 10.47852/bonviewMEDIN32021963

[14] Doehner W Boriani G Potpara T Blomstrom-Lundqvist C Passman R Sposato LA Atrial fibrillation burden in clinical practice, research, and technology development: a clinical consensus statement of the European Society of Cardiology Council on Stroke and the European Heart Rhythm Association. Europace. (2025) 27(3):euaf019. 10.1093/europace/euaf019 40073206 PMC11901050

[15] Noubiap JJ Feteh VF Middeldorp ME Fitzgerald JL Thomas G Kleinig T A meta-analysis of clinical risk factors for stroke in anticoagulant-naïve patients with atrial fibrillation. Europace. (2021) 23(10):1528–38. 10.1093/europace/euab087 34279604

[16] Kim BJ Lee KJ Park EL Tanaka K Koga M Yoshimura S Prediction of recurrent stroke among ischemic stroke patients with atrial fibrillation: development and validation of a risk score model. PLoS One. (2021) 16(10):e0258377. 10.1371/journal.pone.0258377 34624070 PMC8500448

[17] Joglar JA Chung MK Armbruster AL Benjamin EJ Chyou JY Cronin EM 2023 ACC/AHA/ACCP/HRS guideline for the diagnosis and management of atrial fibrillation: a report of the American College of Cardiology/American Heart Association joint committee on clinical practice guidelines. Circulation. (2024) 149(1):e1–156. 10.1161/cir.0000000000001193 38033089 PMC11095842

[18] Hoh BL Ko NU Amin-Hanjani S Chou S-Y Cruz-Flores S Dangayach NS 2023 Guideline for the management of patients with aneurysmal subarachnoid hemorrhage: a guideline from the American Heart Association/American stroke association. Stroke. (2023) 54(7):e314–70. 10.1161/str.0000000000000436 37212182

[19] Heidenreich PA Bozkurt B Aguilar D Allen LA Byun JJ Colvin MM 2022 AHA/ACC/HFSA guideline for the management of heart failure: a report of the American College of Cardiology/American Heart Association joint committee on clinical practice guidelines. Circulation. (2022) 145(18):e895–1032. 10.1161/cir.0000000000001063 35363499

[20] Virani SS Newby LK Arnold SV Bittner V Brewer LC Demeter SH 2023 AHA/ACC/ACCP/ASPC/NLA/PCNA guideline for the management of patients with chronic coronary disease: a report of the American Heart Association/American College of Cardiology joint committee on clinical practice guidelines. Circulation. (2023) 148(9):e9–119. 10.1161/cir.0000000000001168 37471501

[21] Kataoka N Imamura T. Efficacy and feasibility of catheter ablation in elderly patients with atrial fibrillation. Clin Cardiol. (2024) 47(2):e24195. 10.1002/clc.24195 37970723 PMC10823451

[22] Hashim U Patel R Demo H Fisher W Nazari J Ro A Patient comfort and response pattern following pulsed-field ablation compared to radiofrequency ablation for atrial fibrillation. J Cardiovasc Electrophysiol. (2025) 36(11):2950–4. 10.1111/jce.70077 40908575

[23] Tabaja C Hight N Younis A Jadam S Demian J Hussein A Vascular access complications after catheter ablation of ventricular arrhythmias: impact of vascular closure devices. Heart Rhythm. (2025) 22(3):685–92. 10.1016/j.hrthm.2024.09.001 39245246 PMC11875950

[24] Abdur Rehman K Wazni OM Barakat AF Saliba WI Shah S Tarakji KG Life-Threatening complications of atrial fibrillation ablation: 16-year experience in a large prospective tertiary care cohort. JACC Clin Electrophysiol. (2019) 5(3):284–91. 10.1016/j.jacep.2018.11.013 30898229

[25] Padala SK Gunda S Sharma PS Kang L Koneru JN Ellenbogen KA. Risk model for predicting complications in patients undergoing atrial fibrillation ablation. Heart Rhythm. (2017) 14(9):1336–43. 10.1016/j.hrthm.2017.04.042 28479516

[26] Mo P Fan C Chen J Wang Y Xiao W Peng Z Atrial fibrillation types and chronic kidney disease are independent predictors of atrial fibrillation recurrence after radiofrequency ablation. Ther Clin Risk Manag. (2024) 20:817–28. 10.2147/tcrm.S492265 39650859 PMC11624671

[27] Fan W Wei C Liu Y Sun Q Tian Y Wang X The prognostic value of hematologic inflammatory markers in patients with acute coronary syndrome undergoing percutaneous coronary intervention. Clin Appl Thromb Hemost. (2022) 28:10760296221146183. 10.1177/10760296221146183 36567485 PMC9806387

[28] Guan YZ Yin RX Zheng PF Liu CX Wei BL Deng GX. Association of RDW, NLR, and PLR with atrial fibrillation in critical care patients: a retrospective study based on propensity score matching. Dis Markers. (2022) 2022:2694499. 10.1155/2022/2694499 35669502 PMC9166973

[29] Gabet A Chatignoux E Grave C Vallée A Tuppin P Béjot Y Stroke incidence and death in atrial fibrillation patients newly treated with direct oral anticoagulants. Clin Epidemiol. (2021) 13:131–40. 10.2147/clep.S290707 33642879 PMC7903960

[30] Chen X Gu J Zhang X. Brain-Heart axis and the inflammatory response: connecting stroke and cardiac dysfunction. Cardiology. (2024) 149(4):369–82. 10.1159/000538409 38574466 PMC11309082

[31] Battaglini D Robba C Lopes da Silva A Dos Santos Samary C Leme Silva P Dal Pizzol F Brain-heart interaction after acute ischemic stroke. Crit Care. (2020) 24(1):163. 10.1186/s13054-020-02885-8 32317013 PMC7175494

[32] Liu H Lin M Han W Ge J Maduray K Zhong J. The risk factors of thrombus formation and the effect of catheter ablation on repetitive thrombus formation in patients with atrial fibrillation: a single center retrospective study in China. BMC Cardiovasc Disord. (2023) 23(1):28. 10.1186/s12872-023-03050-z 36650447 PMC9843887

[33] Botto GL Tortora G Casale MC Canevese FL Brasca FAM. Impact of the pattern of atrial fibrillation on stroke risk and mortality. Arrhythm Electrophysiol Rev. (2021) 10(2):68–76. 10.15420/aer.2021.01 34401178 PMC8335885

[34] Nedios S Lindemann F Heijman J Crijns H Bollmann A Hindricks G. Atrial remodeling and atrial fibrillation recurrence after catheter ablation: past, present, and future developments. Herz. (2021) 46(4):312–7. 10.1007/s00059-021-05050-1 34223914

[35] Llorente-Chávez A Martín-Nares E Núñez-Álvarez C Hernández-Molina G. Thrombosis and thrombocytopenia in antiphospholipid syndrome: their association with mean platelet volume and hematological ratios. Thromb Res. (2021) 203:12–7. 10.1016/j.thromres.2021.04.018 33895567

[36] Kim YG Shim J Oh SK Lee KN Choi JI Kim YH. Risk factors for ischemic stroke in atrial fibrillation patients undergoing radiofrequency catheter ablation. Sci Rep. (2019) 9(1):7051. 10.1038/s41598-019-43566-z 31065030 PMC6504925

[37] Rathfoot C Edrissi C Sanders CB Knisely K Poupore N Nathaniel T. Gender differences in comorbidities and risk factors in ischemic stroke patients with a history of atrial fibrillation. BMC Neurol. (2021) 21(1):209. 10.1186/s12883-021-02214-8 34034655 PMC8146651

[38] Saliba W Schliamser JE Lavi I Barnett-Griness O Gronich N Rennert G. Catheter ablation of atrial fibrillation is associated with reduced risk of stroke and mortality: a propensity score-matched analysis. Heart Rhythm. (2017) 14(5):635–42. 10.1016/j.hrthm.2017.02.001 28189823

